# Removal of back-reflection noise at ultrathin imaging probes by the single-core illumination and wide-field detection

**DOI:** 10.1038/s41598-017-07024-y

**Published:** 2017-07-26

**Authors:** Changhyeong Yoon, Munkyu Kang, Jin H. Hong, Taeseok D. Yang, Jingchao Xing, Hongki Yoo, Youngwoon Choi, Wonshik Choi

**Affiliations:** 1 Center for Molecular Spectroscopy and Dynamics, Institute for Basic Science, Seoul, 02841 Korea; 2 Department of Physics, Korea University, Seoul, 02841 Korea; 3 Department of Bioengineering, Hanyang University, Seoul, 04763 Korea; 4 School of Biomedical Engineering, Korea University, Seoul, 02841 Korea

## Abstract

Thin waveguides such as graded-index lenses and fiber bundles are often used as imaging probes for high-resolution endomicroscopes. However, strong back-reflection from the end surfaces of the probes makes it difficult for them to resolve weak contrast objects, especially in the reflectance-mode imaging. Here we propose a method to spatially isolate illumination pathways from detection channels, and demonstrate wide-field reflectance imaging free from back-reflection noise. In the image fiber bundle, we send illumination light through individual core fibers and detect signals from target objects through the other fibers. The transmission matrix of the fiber bundle is measured and used to reconstruct a pixelation-free image. We demonstrated that the proposed imaging method improved 3.2 times on the signal to noise ratio produced by the conventional illumination-detection scheme.

## Introduction

Endoscopy enables us to visualize objects whose line of sight is blocked by intervening layers. This has made it an essential disease diagnosis tool in the biomedical field. For example, flexible endoscopes are used for examining the superficial structures of internal organs such as colons and stomachs. Rigid-type endoscopes such as laparoscopes and arthroscopes can usefully provide surgical guidance with clear visibility. Recent technological developments have enhanced the spatial resolution of endoscopes to close to that of optical microscopes and reduced the diameters of the imaging probes to below a millimeter. The so-called endomicroscopes satisfying these two requirements provide a minimally invasive way of investigating the fine details of the microenvironments within target organs. Since sophisticated high-resolution optical systems are hard to reduce to such small dimensions, graded-index (GRIN) rod lenses or image fiber optic bundles are often used as imaging probes instead^1–6^. Despite being simple in structure, these probes support the high numerical aperture (NA) necessary for microscopic imaging.

When using such ultrathin imaging probes, the same probe is used for both illumination and collection to limit the dimensions of the endoscope unit to the diameter of the probes. Consequently, light reflected from the surface of the probe facing the target object is inevitably detected at the image sensor. This back-reflection from the distal end of the probe overlaps with the signal light backscattered from target objects. Since the contrast of biological tissues is weak, back-reflected light can degrade the signal to noise ratio to the extent that biological targets become invisible. As a point of comparison, macroscopic-scale imaging by typical endoscopes, a method subject to less severe size restriction, avoids this issue by introducing illuminating light through separate fibers.

In fact, back-reflection noise is not a big issue for fluorescence imaging as it can simply be filtered out of the fluorescence signal by spectral filters. For this reason, fluorescence-based endomicroscopes have been successfully developed in many previous studies^1, 7–11^. However, most fluorescence imaging modalities require labeling agents and are thus limited to use in animal studies and *in vitro* specimens. On the other hand, the reflectance-mode of imaging, which is directly applicable to *in vivo* studies in biomedical applications, is extremely susceptible to back-reflection noise. Since in this case the wavelengths of illumination and detection are the same, back-reflection noise from imaging probes is indistinguishable from signal light. The simplest way to avoid back-reflection noise is to deliver illumination light through a separate beam path. However, this requires a long working distance, thereby reducing the achievable NA. The use of oil immersion can greatly reduce back-reflection noise, but may not be an option for most *in vivo* applications. The time gating method used in optical coherence tomography helps to reduce back-reflection noise as the optical path length of the signal light differs from that of the noise. In any case, the strong back-reflection noise still wastes the dynamic range of the detectors, thereby undermining their ability to resolve weak contrast targets.

In this Letter, we present a rigid-type endomicroscope free from back-reflection noise originating from the distal end of the thin imaging probes, and yet capable of performing high-resolution and wide-field reflectance imaging. To physically separate the illumination and imaging paths, an image fiber bundle is used as an imaging probe with no lens attached at the distal end. We then employ a unique illumination-detection geometry in which the illumination light is delivered through individual core fibers of the bundle, one by one, and the signal light from the target object is collected by other fibers. The transmission matrix describing the optical response of the fiber bundle is used to reconstruct the object image from the signals at the other fibers at a spatial resolution of 1.0 μm^12, 13^. The resulting signal to noise ratio (SNR) was 3.2 times better than that obtained with conventional wide-area illumination approaches. We demonstrated the imaging of rat intestines exposed to air where strong back-reflection noise is usually introduced.

## Methodology

**Figure 1:**
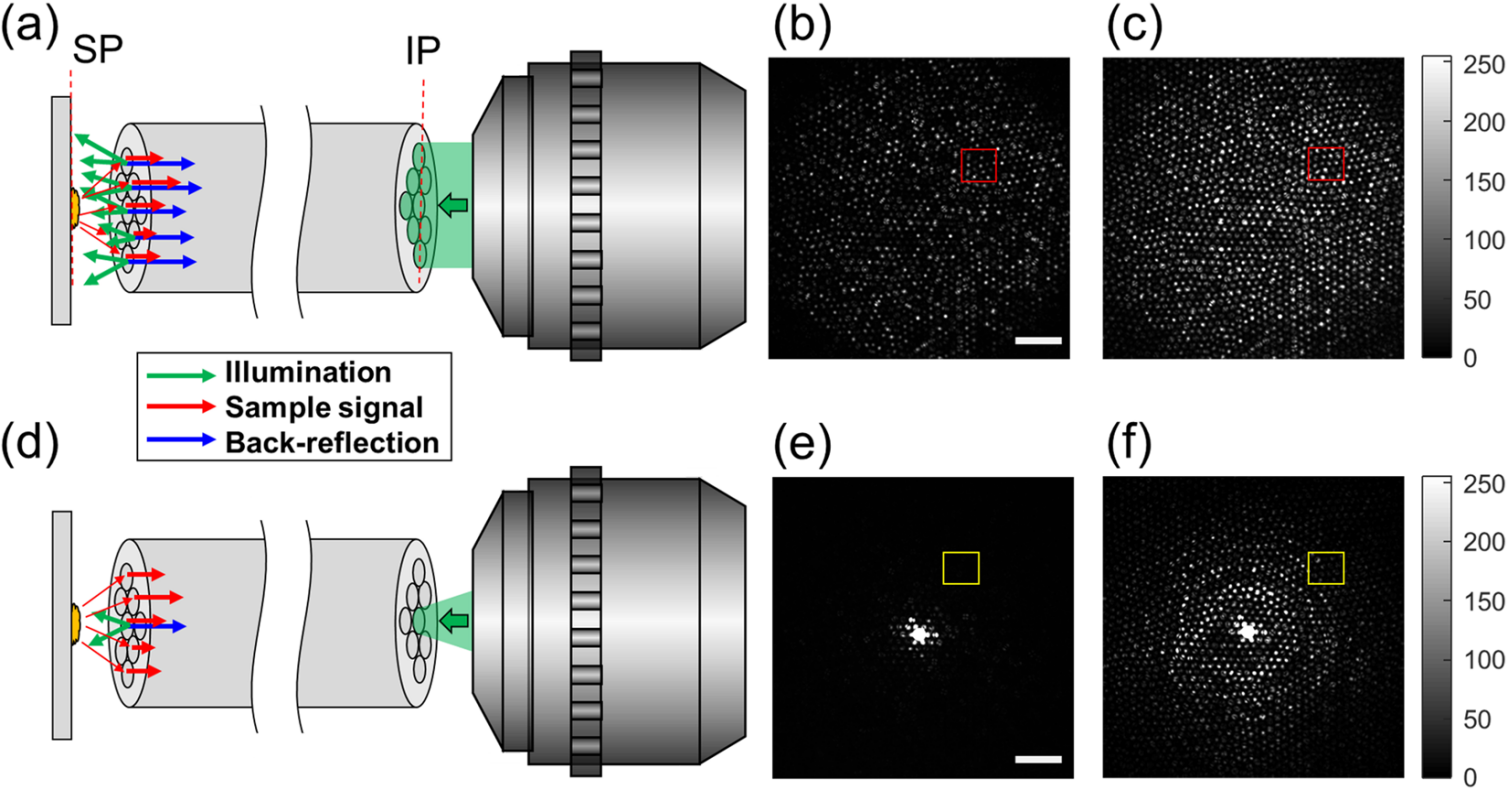
Schematic layouts of the WIWD and SIWD detection. (**a**) The conventional wide-field illumination and wide-field detection (WIWD) method. Green shading and green arrows indicate illumination. Blue and red arrows represent back-reflection noise at the distal end of the fiber and the backscattered light from the target object, respectively. IP: illumination plane, SP: sample plane. (**b**,**c**) Intensity images recorded at the IP without and with a target object, respectively. Scale bar: 20 μm. Color bar: intensity in arbitrary units. The color scales of (**b**,**c**) are the same. (**d**) The single-core illumination and wide-field detection (SIWD) method. (**e**,**f**) Same as (**b**) and (**c**), respectively, but for the SIWD method. Scale bar: 20 μm. Color bar: intensity in arbitrary units. Color scales of (**e**) and (**f**) are the same.

In the conventional wide-field illumination and wide-field detection (WIWD) method, all the core fibers in the bundle are used for both irradiating an object and collecting the backscattered light from the object (Fig. 1a). Since the illumination and detection pathways are the same, back-reflection light from the surface of the individual core fibers near the sample plane (SP) are always superimposed on the signal light from the object. In fact, the same is true of the single-core illumination and single-core detection scheme used by scanning fluorescence endomicroscopes. As shown in Fig. 1b, an image taken at the illumination plane (IP), all fiber cores had returning signals even when there was no target object in the SP. Figure 1c is representative of the images recorded at the IP when a 1951 United States Air Force (USAF) resolution target (#58-198, Edmund Optics) was placed at the SP. The back-reflection noise shown in Fig. 1b already contributed to this image and degraded the SNR.

In contrast, the single-core illumination and wide-field detection (SIWD) method that we propose delivers illumination light through single individual core fibers and the signal is acquired via the other core fibers (Fig. 1d). Figure 1e shows the reflection noise taken at the IP in the absence of a target object. Back-reflection noise was only present at the core used for illumination and some of its neighbors. The returning signal is dispersed across the other fibers, as shown in the image taken at the SP after insertion of the USAF target (Fig. 1f). In fact, the SP was not in direct contact with the fiber bundle, but at a distance D = 100 μm. Thus, the light from the single core can spread to illuminate a wide sample area, and the returning light from the object is distributed across the other fiber cores. Since the intensity of the reflection noise is highly localized, it can be easily discriminated and eliminated by excluding the signal from the illumination core and its neighbors. Consequently, the remaining cores carry no reflection noise, and thus in the SIWD method the full dynamic range of the detector can be used to capture the signals from the samples. We verified the noise rejection capability of SIWD by calculating its signal to background ratio. When using WIWD, I_s_/I_b_ was found to be about 2, where I_s_ and I_b_ are the average intensities in the square boxes in Fig. 1b and c, respectively. When using SIWD, the I_s_/I_b_ was found to be greater than 10. Therefore, the detected signal to background ratio increased more than 5 times.

One important requirement for the application of SIWD is that the SP is not in contact with the fiber bundle, but a distance away. More generally, the direct image relation between the surface of the fiber bundle and the SP needs to be broken. Otherwise, the illumination would not spread out across the object, so the signal light scattering from the object would only return through the same core fiber used for illumination. Since the SP is not at the conjugate plane of the surface of the fiber, signals captured by the other fibers do not form an image. To recover the original object image from the signals detected at the other fibers, a transmission matrix approach previously developed by us was used^12–16^. In brief, we measured the transmission matrix describing the input-output response of the fiber bundle from SP to IP, and multiplied the inversion of the matrix ﻿to the images recorded at the IP﻿﻿ to recover the object image at the SP.

## Experimental setup

**Figure 2:**
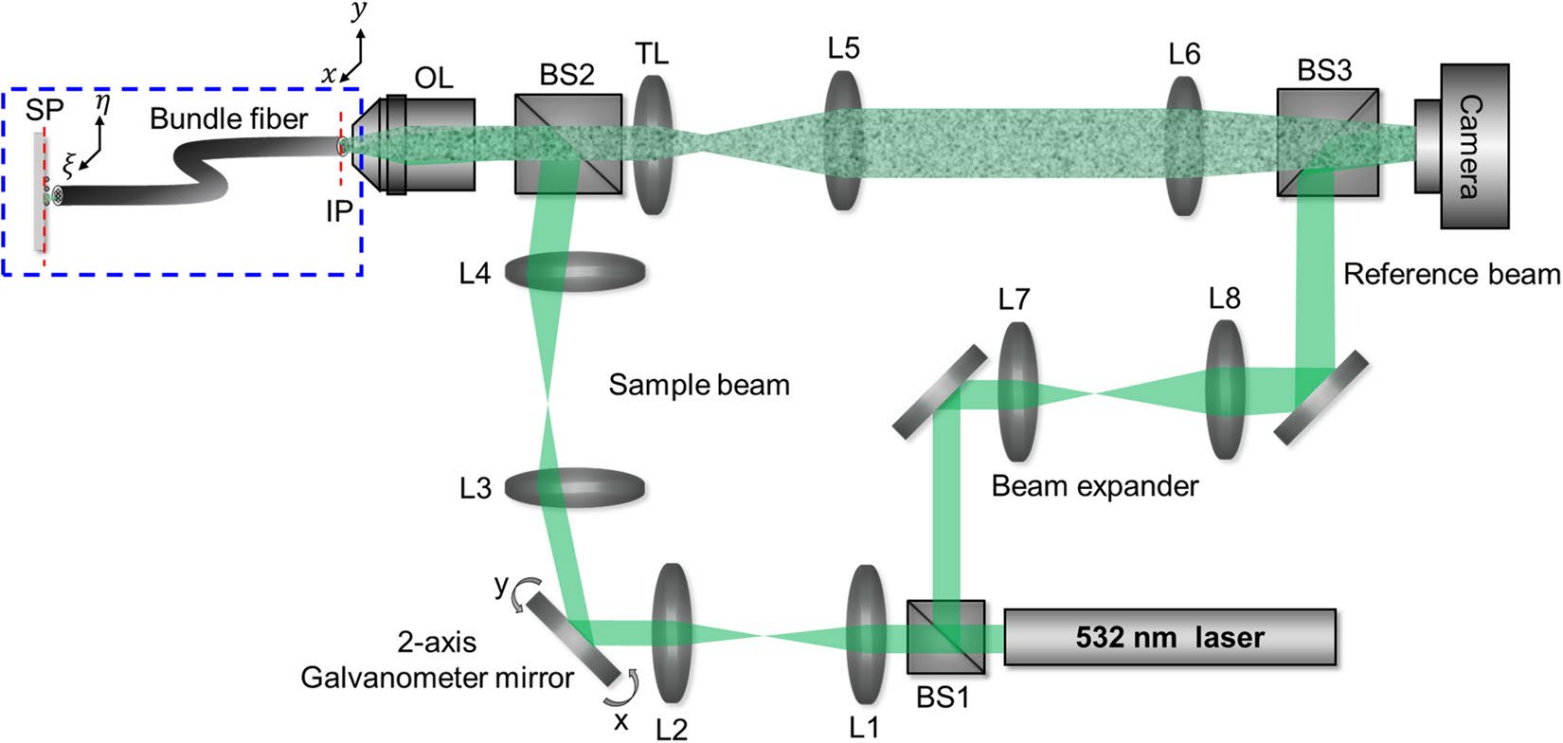
Schematic layout of the experimental setup. Interference microscope with a 2-axis scanning mirror installed in the sample bream path. A target object is placed at the SP. For clarity, the spatial coordinates at IP and SP are denoted as (*x,y*) and (ξ,η). BS1-3: beam splitters, L1-8: lenses, OL: objective lens, TL: tube lens.

**Figure 3:**
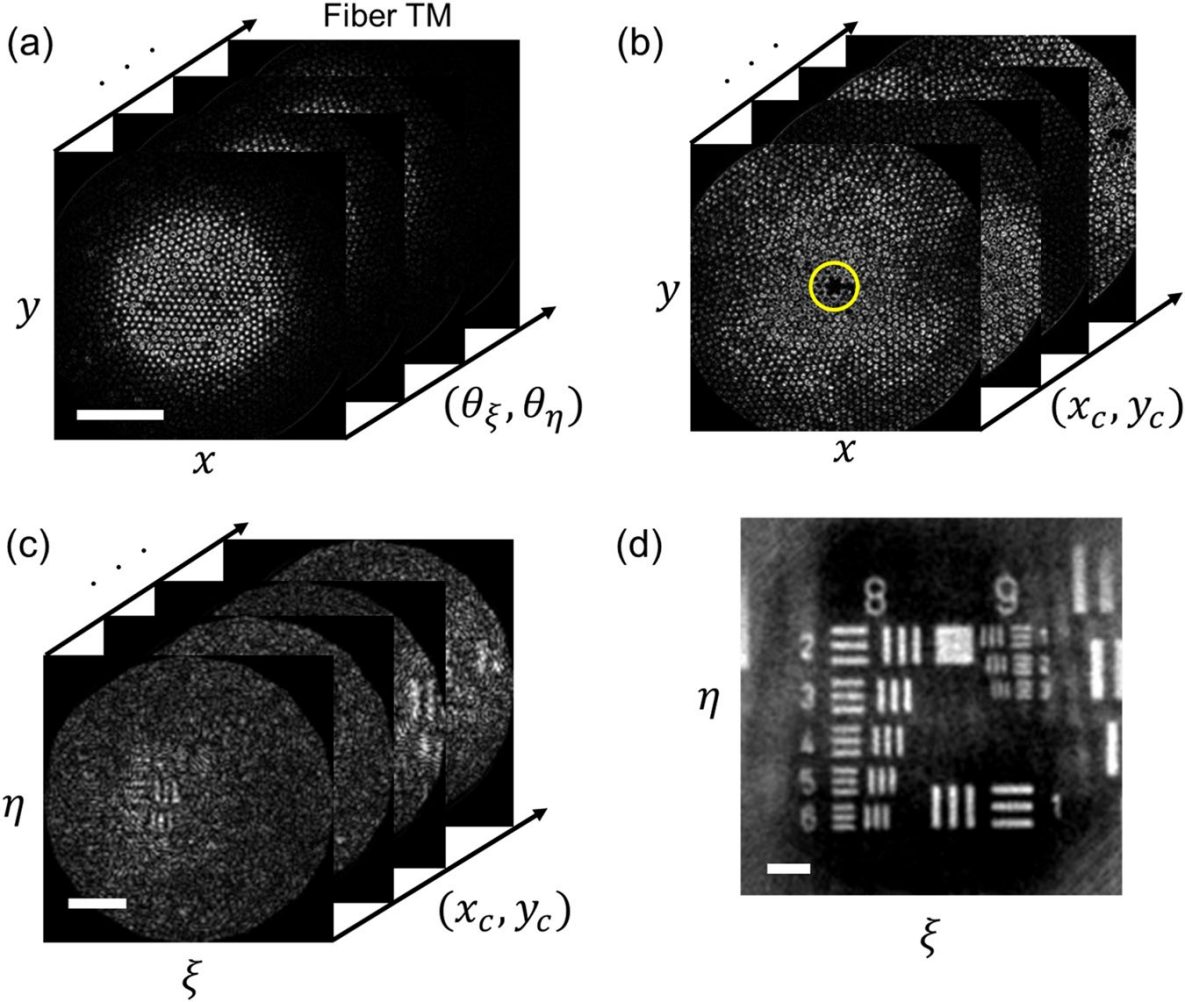
Image acquisition using the single-core illumination and wide-field detection scheme. (**a**) Transmission matrix of the fiber bundle. Complex field maps were recorded at (*x,y*) for the scanning of illumination angle (*θ*_*ξ*_,θ_η_) at the (*ξ,η*) plane. Scale bar: 50 μm. (**b**) Recording of sample images at (*x,y*) with focused illumination from individual fiber cores (*x*_*c*_,y_c_). The dark spot indicated by a yellow circle is the core fiber through which the illumination was focused. (**c**) Object images reconstructed by multiplying each of the individual images in (**b**) with the inverse of the transmission matrix in (a). Scale bar: 20 μm. (**d**) Incoherent summation of all the images in (**c**). Scale bar: 10 μm.

We constructed an experimental SIWD setup based on a reflection-type interferometric microscope with an off-axis detection configuration (Fig. 2). A diode laser (finesse pure pump laser, Laser Quantum) with a wavelength of 532 nm was used as a light source. The collimated laser output was split into sample and reference beams by a beam splitter (BS1). The sample beam was delivered to the back aperture of an objective lens (Olympus, PlanN, 20X, 0.4 NA) by two 4-*f* telescopes consisting of lenses (L1–4) via another beam splitter (BS2), such that the sample beam was focused on the IP. A 2-axis galvanometer scanning mirror (GVS211, Thorlabs) was placed at a Fourier plane between the two 4-*f* telescopes to scan the point of illumination at the IP. An image fiber bundle (Fujikura, FIGH-06-300S, 0.8 m long, 300 μm diameter, numerical aperture *α*=0.4) comprised of 6,000 core fibers with 2.2 μm in diameter was used as the imaging probe. The focused light was coupled to single cores of the fiber bundle at the IP and at the opposite side of the fiber illuminated an object located at the SP. The SP was about D = 100 μm away from the surface of the fiber bundle so that an object was irradiated over a wide area rather than a single point. The area of illumination is given by 2*αD≈*80 μm. The captured light was guided to the IP, delivered to a camera and combined with the reference beam at the beam splitter (BS3) to form an interference pattern on the camera (sCMOS, pco.edge 4.2, PCO). To obtain the reliable contrast of the interference, the optical path length difference between the sample and the reference arms was set to be smaller than the 4 mm-coherence length of the laser. The stability of the interferometer was measured to be about 70 mrad and 1 rad in a short-term (1 second) and a long-term (120 seconds) period, respectively. This phase noise occurring during the experiments was numerically traced and compensated in the image processing. The recorded interferograms were processed into complex field images, providing amplitude and phase maps simultaneously. We repeated the measurements while adjusting the angles of the scanning mirrors such that the focused spot illuminated single cores of the fiber bundle at IP. As pointed out, the image recorded at the camera does not represent the actual object image as the distal end of the fiber bundle was about 100 μm away from the SP. To allow recovery of the object image, we measured the transmission matrix of the fiber bundle from the SP to the IP as a one-time calibration procedure. We sent a plane wave of incidence angle (*θ*_*ξ*_,*θ*_*η*_) through the SP using an additional illumination port (not shown in Fig. 2) and measured the complex field map *E*(*x*,*y*;*θ*_*ξ*_,*θ*_*η*_) at the IP (Fig. 3a). We repeated these measurements while scanning the illumination angle at SP. After converting each image into a one-dimensional column vector, we combined all the column vectors to form the matrix *t*(*x*,*y*;*θ*_*ξ*_,*θ*_*η*_), a transmission matrix describing the input-output response of the fiber bundle. To create the transmission matrix we typically took 6,000 maps covering up to 0.3 NA, which sets the diffraction-limit of reconstructed images to be 1.0 μm.

After acquiring the transmission matrix, a target object was placed at SP for imaging. The 2-axis galvanometer mirror was tuned to illuminate each core of the fiber located at (*x*_*c*_,*y*_*c*_), and the complex field map *E*_*S*_(*x*,*y*;*x*_*c*_,*y*_*c*_) returning from the target to the IP was taken by the camera. Typically, 1,800 sample images were taken corresponding to the illumination of as many fiber cores (Fig. 3b). The image acquisition took about 36 seconds due to the speed limit of the camera used in the experiment. This can be reduced to 1 s or less if a high-speed camera is used. Once a set of reflection images of an object had been taken, the object image could be reconstructed based on the turbid lens imaging (TLI) method combined with speckle averaging^12, 17, 18^. Specifically, we multiplied the inverse of the transmission matrix (Fig. 3a) to each individual object image in Fig. 3b to obtain the angular spectrum map of the target at SP.

1 $${E}_{S}({\theta }_{\xi },{\theta }_{\eta };{x}_{c},{y}_{c})=t{(x,y;{\theta }_{\xi },{\theta }_{\eta })}^{-1}{E}_{S}(x,y;{x}_{c},{y}_{c}).$$

From the acquired angular spectrum map, we reconstructed the object image *E*_*S*_(*ξ*,*η*;*x*_*c*_,*y*_*c*_) for the illumination through each core (Fig. 3c). This operation removes the image distortion caused by the distance D as well the pixelation caused by the multiple cores of the fiber bundle. However, the reconstructed object images were still unclear since the illumination through individual core fibers generated speckled fields at the SP. To reduce this speckle noise, individually reconstructed images were added incoherently to produce a clean object image (Fig. 3d). The spatial resolution was 1.0 μm, which was in good agreement with the diffraction limit given by the numerical aperture of the transmission matrix. Since we measure the complex field images of an object, we can numerically adjust the focal plane for the image reconstruction within a range of about ± 70 µm from the original focus. Thus, the volumetric data for the thickness of 140 µm can be attainable from a single focus image without scanning the imaging probe (See Supplementary Movie S1). For the overall imaging processing, it takes about 120 seconds for the generation of a transmission matrix, and 5 minutes to produce the final object image. This can be shortened by employing more efficient reconstruction algorithm or simply by using faster computers.

## Results

**Figure 4:**
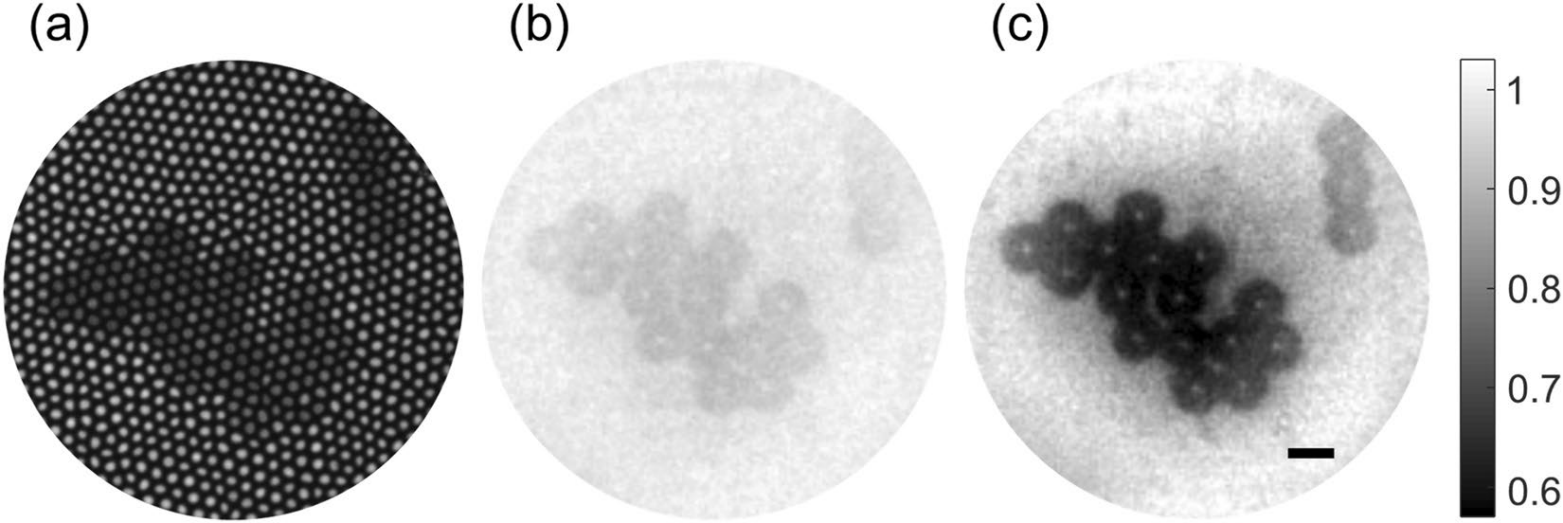
Imaging polystyrene beads in air. (**a**) Conventional reflection imaging with LED illumination, (**b**) with WIWD method, (**c**) with SIWD method. Scale bar: 10 μm. For both (**b**) and (**c**), the background intensity was normalized and the color scales are the same. Color bar: normalized intensity.

We conducted an experiment to compare the performances of the SIWD and WIWD methods. A test sample of polystyrene beads (Sigma-Aldrich, 10 μm diameter) was mounted on a slide and imaged by both methods. No immersion medium was used between the fiber bundle and the beads so that the strong back-reflection noise arose from the distal end of the fiber bundle. First, we observed a bead cluster with a conventional reflection imaging configuration in which LED illumination was sent from IP. In this case, the distal end of the fiber bundle was brought into contact with the bead cluster so that the IP was in conjugate plane to the SP. The resulting recorded image was highly pixelated as shown in Fig. 4a. Considering that the cores of the fiber bundle are 2.2 μm in diameter and their separation is about 3.4 μm, the beads, which have a diameter of 10 μm, should be resolved. However, they were almost invisible due to the degradation of image contrast induced by strong back-reflection at the surface of the fiber bundle.

In the WIWD method, we sent collimated planar waves to illuminate the entire cores of the bundle at IP. For a fair comparison with SIWD, 1,800 object images were taken while varying the angles of illumination at IP. The object image was reconstructed following the procedure shown in Fig. 3 and, while the resulting image (Fig. 4b) allows the overall shape of the bead cluster to be observed, it still suffered from extremely poor contrast. However, the image acquired by the SIWD method (Fig. 4c) clearly visualized all the beads with much better contrast, mainly due to the rejection of back-reflection noise. For a quantitative comparison, we defined the signal as the difference in intensity between the background and the target, and noise as the standard deviation of the background. The measured signal to noise ratio was 3.1 times larger in the case of SIWD than that of WIWD.

Finally, we demonstrate the ability of SIWD scheme to image a biological tissue when no immersion medium is present between the imaging probe and the tissue. Typically, the reflection signal from biological tissues is much weaker than the back-reflection noise from the distal end of the imaging probe. Although immersion media such as water or greasy materials are often used to reduce back-reflection noise by reducing the index mismatch at the interface, they are inapplicable to most *in vivo* applications. Since the SIWD scheme can selectively reject back-reflection noise, it can image biological tissues to a high SNR without the application of an immersion medium. For a test sample, we extracted intestine tissue from a 2- or 3-day-old rat, and fixed it by the standard fixation protocol using formaldehyde. After being stored in a 4% formaldehyde solution overnight at room temperature for stabilization, the intestine tissue was washed with Hank’s balanced salt solution (HBSS) and then exposed to air for imaging. Since the sizes of individual villi were greater than the field of view of the fiber bundle, images of multiple sites were acquired by translating the tissue and then mosaicked to produce the final images.

**Figure 5:**
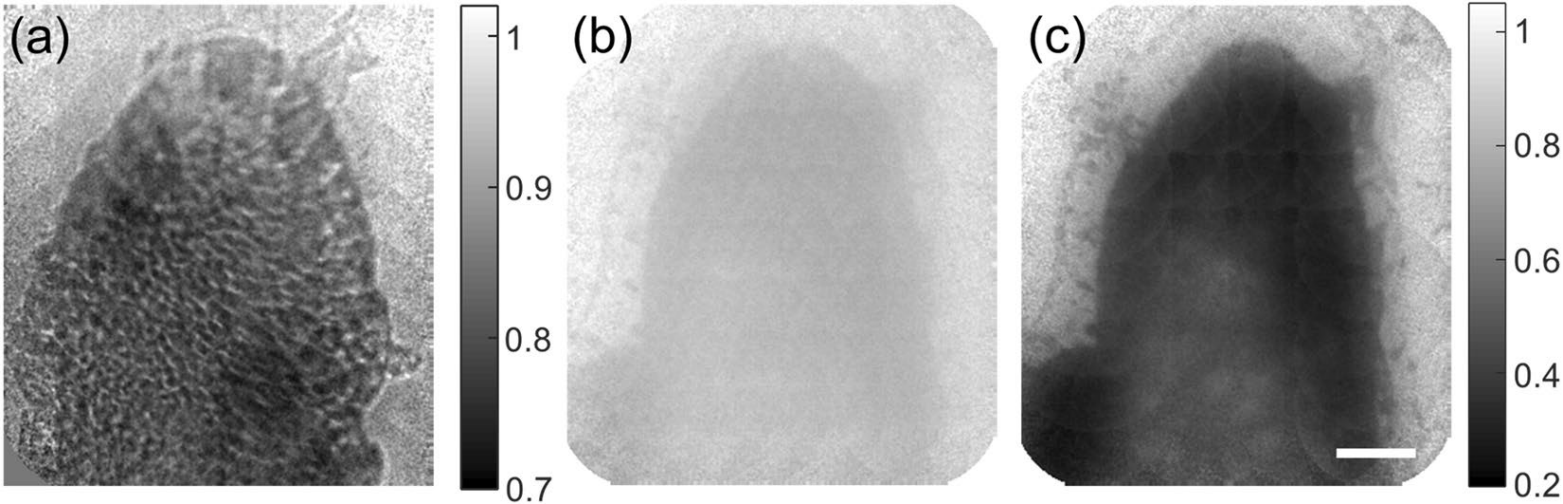
*Ex vivo* imaging of a small villus of a rat intestine. (**a**) WIWD in oil, (**b**) WIWD in air, and (**c**) SIWD in air. Scale bar: 50 μm. For (**b**) and (**c**), the background intensity was normalized, and the color scales are the same. Color bars: normalized intensity.

As a point of reference, we took the image of the villus with the WIWD method with immersion oil placed between the specimen and the fiber bundle (Fig. 5a). Due to the reduction in reflection noise on replacing the air-fiber interface with an oil-fiber interface, the villus was clearly resolved. Figure 5b shows a villus taken by the WIWD method when there was no immersion oil. The overall shape is barely resolved due to the poor SNR caused by the increase in back-reflection noise. Next, we imaged the same villus with the SIWD method (Fig. 5c), and observed that the SNR was 3.2 times better than that with the WIWD method. The shape of the villus is clearly seen even though the specimen was exposed to the air. This confirms that the SIWD method has a comparable performance with the index matching method of conventional WIWD, but without the application of any immersion material.

## Discussion

In conclusion, we have developed an endoscopic imaging method free from back-reflection noise generated at an ultrathin imaging probe and yet guaranteeing microscopic spatial resolution. We employed a unique illumination-detection scheme to spatially separate signal waves from back-reflection noise. In our method, the illumination was delivered through single core fibers in the image fiber bundle, but the signal waves were detected by the other core fibers. By blocking the back-reflection, which occurred only at the core used for the illumination, we could remove the back-reflection noise before it reached the detector sensor. Since this unique imaging scheme requires a target object to be placed at a distance from the surface of the fiber bundle, the signals detected at the other core fibers cannot form an object image. To solve this problem, we experimentally measured the transmission matrix of the fiber bundle and successfully reconstructed an object image from the signals collected by the other core fibers. By systematically assessing the performance of the proposed method, we found that the signal to noise ratio it achieved was more than three times larger than that using the conventional illumination-detection scheme. We also showed that the new method could image a biological tissue exposed to air and achieve an enhanced contrast comparable to that achieved by the conventional illumination configuration, but without using an immersion medium. Since the proposed method requires the use of a transmission matrix for image reconstruction, it can be employed to the rigid-type endomicroscope. If a fiber bundle is housed within a metal needle, its transmission matrix can stay invariant even with the scanning of the probe. The immersion-free feature of our method will facilitate the application of thin endomicroscopic probes to a wide variety of *in vivo* imaging scenarios. The proposed method can also be combined with other reflectance imaging modalities, such as endoscopic optical coherence imaging, and may thereby also improve the sensitivities of these other imaging modalities.

## Electronic supplementary material

Supplementary Movie S1

Supplementary Movie S1 caption
