# Supplementary material for: Removal of back-reflection noise at ultrathin imaging probes by the single-core illumination and wide-field detection
Source: Sci Rep. 2017 Jul 26;7:6524. doi: 10.1038/s41598-017-07024-y (PMC5529376; doi:10.1038/s41598-017-07024-y)
Supplement: Supplementary file 2 — Supplementary Movie S1 caption [file 41598_2017_7024_MOESM2_ESM.pdf]

Removal of back-reflection noise at ultrathin imaging probes by the single-core illumination and wide-field detection

Changhyeong Yoon[1,2], Munkyu Kang[1,2], Jin H. Hong[1,2], Taeseok D. Yang[4], Jingchao Xing[3], Hongki Yoo[3], Youngwoon Choi[4,*], and Wonshik Choi[1,2,*]

[1]Center for Molecular Spectroscopy and Dynamics, Institute for Basic Science, Seoul 02841, Korea
[2]Department of Physics, Korea University, Seoul 02841, Korea
[3]Department of Bioengineering, Hanyang University, Seoul 04763, Korea
[4]School of Biomedical Engineering, Korea University, Seoul 02841, Korea
Corresponding author: youngwoon@korea.ac.kr, wonshik@korea.ac.kr

Movie S1. Numerical propagation of the focal plane for the image reconstruction. The numerical refocusing was performed from -70 μm to +70 μm around the center where the original focus of the image acquisition was located. The sample is clusters of polystyrene beads with 10 μm diameter. During the numerical scanning two different bead clusters were in focus at -50 μm and +50 μm. Scale bar: 10 μm
